# Role of H_3_O· Radical in the Degradation of Fuel Cell Proton-Exchange Membranes

**DOI:** 10.1021/acsphyschemau.2c00037

**Published:** 2022-10-18

**Authors:** Hai Long, Clara Larson, Frank Coms, Bryan Pivovar, Gregg Dahlke, Michael Yandrasits

**Affiliations:** † Computational Science Center, National Renewable Energy Laboratory, 15013 Denver West Parkway, Golden, Colorado80401, United States; ‡ Global Fuel Cell Business, General Motors Company, 850 N Glenwood Avenue, Pontiac, Michigan48340, United States; § Chemical and Materials Science Center, National Renewable Energy Laboratory, 15013 Denver West Parkway, Golden, Colorado80401, United States; ∥ 3M Advanced Materials Division Laboratory, 3M Center, Saint Paul, Minnesota55144-1000, United States; ⊥ 3M Corporate Research Materials Laboratory, 3M Center, Saint Paul, Minnesota55144-1000, United States

**Keywords:** proton-exchange membrane fuel cell, membrane degradation, hydronium radical, radical reaction mechanism, ab initio modeling, ion-radical interaction

## Abstract

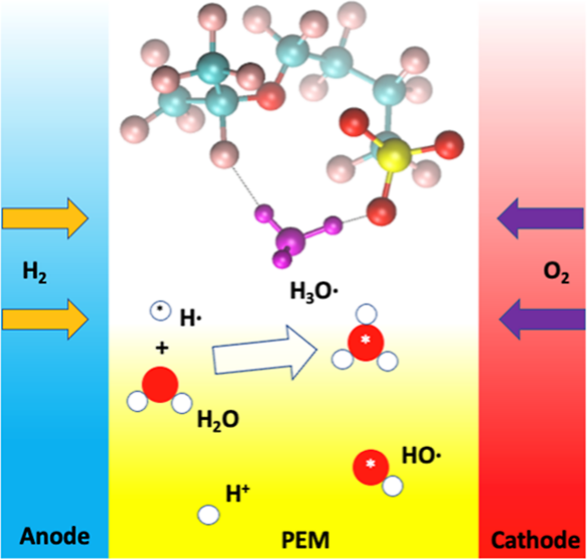

Membrane durability in proton-exchange membrane fuel cells (PEMFCs) is one of the major obstacles limiting its applications, especially in heavy-duty vehicles. Membrane degradation reactions are thought to be attacks by radicals such as hydroxyl (HO^•^) or hydrogen atom (H^•^) generated during fuel cell operation. For the H^•^ case, computational modeling results have suggested that the reaction between H^•^ and the sulfonic group should be the dominant degradation pathway. However, experimental work implies that the tertiary fluorine (*t*-F) attack is the dominant H^•^ reaction pathway, apparently contradicting the theoretical prediction. Based on previous experimental evidence on isotopic substitution, we postulate that the hydronium radical (H_3_O^•^) might be present in PEMFCs. Our ab initio modeling indicates that this radical can be stabilized by the sulfonic anion on the polymer side chain. With the assistance of explicit water, the polymer side chain can undergo a conformational change, leading to a greatly reduced barrier for the *t*-F degradation reaction. Thus, our H_3_O^•^ hypothesis is able to explain not only the previous isotopic substitution experiment but also why the *t*-F degradation reaction is a highly plausible H^•^ degradation mechanism for proton-exchange membranes. To our knowledge, this is the first suggestion that H_3_O^•^ radicals could be present in electrochemical devices with both experimental and theoretical support.

## 1 Introduction

**Figure 1:**
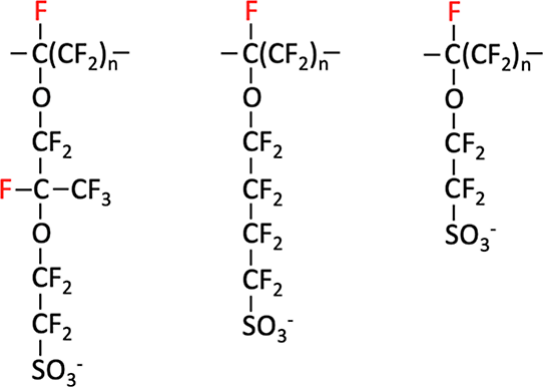
Structures of Chemours Nafion, 3M, and Solvay Aquivion PFSA ionomers. Atoms in red are the tertiary fluorine (*t*-F).

Although perfluorosulfonic acid (PFSA)-based proton-exchange membrane fuel cells (PEMFCs) have long been the most widely used fuel cell (FC) type, membrane durability is still a major obstacle that limits these FCs’ applications, especially in heavy-duty vehicles.^1−5^Figure 1 shows the three most widely used commercial PFSA ionomers in PEMFCs. The proton-exchange membrane (PEM) serves as the gas separator as well as the proton (H^+^) conductor, in that the sulfonic groups of PFSA only allow cations (mainly H^+^) to transport across the membrane. The degradation of PEMs results in not only the loss of sulfonic groups, which reduces the conductivity of the membrane, but also mechanical failure, leading to more gas crossing and further degradation.^1,2^

The membrane degradation mechanisms in PEMFCs are still not completely understood. In general, the degradation reactions are thought to be attacks by radicals generated during FC operation.^1,2^ The presence of radicals has been confirmed by many different experimental techniques. Direct identification of “in situ” radicals has been reported by Schlick et al.^6,7^ using a spin-trapping electron spin resonance technique with 5,5-dimethylpyrroline *N*-oxide (DMPO) as a spin trap. DMPO then reacts with radicals and forms the adduct product via the following reaction
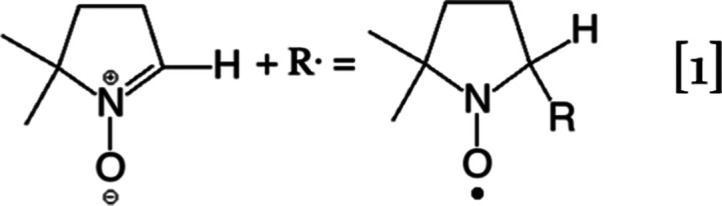
1

Adduct products of DMPO/OH, DMPO/OOH, and DMPO/H have been obtained from the spin trap under different conditions, indicating the possible presence of hydroxyl (HO^•^), hydroperoxyl (HOO^•^), and hydrogen (H^•^) radicals.

Our recent experimental work has suggested that the PFSA polymer chain end unzipping by reactions between ending carboxylate groups and radicals appears to be the dominant degradation mechanism at an open-circuit voltage.^8^ However, because modern PFSA ionomers are already treated to convert the backbone ending into stable −CF_3_ groups, those ending carboxylate groups may be generated from chain cleavage as a result of radical attack reactions on the polymer backbones and side chain. Density functional theory (DFT) calculations from previous reports, as well as in this work, show that these radicals can attack via different reaction pathways.^9−15^ The following degradation reactions are found to have relatively lower degradation barriers (Δ*E*^≠^) and could be the candidates for the dominant degradation pathway for backbone and side chain degradation:^10,12^
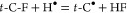
2
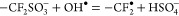
3
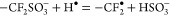
4

In Reaction 2 (Rxn 2), the *t*-C is the tertiary carbon (Figure 1) that links with the polymer side chain. Table 1 summarizes Δ*E*^≠^ values computed using different ab initio methods from previous reports and this work. These data indicate that Rxn 4 has the lowest Δ*E*^≠^ among Rxn 2, 3, and 4. Indeed, the Δ*E*^≠^ of Rxn 4 is almost consistently ∼10 kcal/mol lower than that of Rxn 2 for all DFT methods, and 4∼9 kcal/mol lower than that of Rxn 3 for all DFT methods except the method listed in Row 6 of Table 1. In order to check whether this was caused by the limitations of DFT methods, we also performed MP2 (second order Møller–Plesset perturbation theory)^16^ calculations and still obtained a similar result (Row 8 in Table 1). These results suggest that Rxn 4 should be the dominant H^•^ degradation pathway and would lead to a large amount of sulfur content in the degradation product. Although implicit solvation models such as PCM (polarizable continuum model)^17^ and SMD (solvation model based on density)^18^ are used for the Δ*E*^≠^ values reported in Table 1, we also performed vacuum calculations, and the results still indicated that Rxn 4 should be the dominant pathway. However, very few sulfur compounds are observed experimentally during degradation, contradicting the theoretical prediction.^8,19−21^ This led us to consider the possibility that there might be a hidden radical in PEMFCs that could react with fluorine atoms but escape from the spin trap.

**Table 1:** Δ*E*^≠^ Values in kcal/mol for Rxns 2–4, Computed Using Different Ab Initio Methods^a^

Row	Methods	Rxn 2	Rxn 3	Rxn 4
1	B3LYP/6-311++G(2d,p)/PCM	23.1	22.1	N/A^b^
2	ωB97XD/6-311++G(2d,2p)/PCM	32.1	27.8	23.5
3	B3LYP/6-311G**/C-PCM	22.9	-b	-b
4	B3LYP/6-311++G(2d,p)/PCM	23.6	23.0	13.9
5	ωB97XD/6-311++G(2d,p)/PCM	31.8	26.2	22.3
6	M062X/6-311++G(2d,p)/SMD	31.6	27.3	22.8
7	B97D3/6-311++G(2d,p)/PCM	22.6	11.2	11.5
8	MP2/6-311++G(2d,p)/PCM	39.3	35.1	27.0

a Rows 1, 2, and 3 are from Yu et al.,^10^ Zhao et al.,^12^ and Bajaj et al.,^15^ respectively, and the other rows are from our calculations. Please note that due to the differences in the model compound and whether the zero-point vibrational energy correction (ZPVE) was included, there are small energy differences between literature values and our values, even for the same method. For rows 4–8, no ZPVE is included, and the model compound used is **1** (Figure 2) due to the high computational cost of MP2 calculations.

b “N/A”: No transition state (TS) is found; “-”: not computed.

Another interesting result from the DMPO spin-trap experiment is the presence of DMPO/H adduct in experiments with D_2_ at the anode, with 32% DMPO/D and 68% DMPO/H under close circuit voltage condition before FC operation, and 35 versus 65%, respectively, after 120 min of FC operation.^6^ Thus, the H/D ratio is roughly 2:1, and the H isotope must come from water molecules. This leads us to postulate that there might be a hydronium radical (H_3_O^•^) present in PEMFCs formed by H^•^ reacting with a H_2_O, and it would then react with DMPO as follows
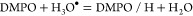
5

The produced H_2_O cannot be detected in experiments, which leads to the very same adduct product of H^•^. However, when D_2_ is introduced at the anode, DH_2_O^•^ might be produced, either by the direct reaction between D_2_ and H_2_O or by a two-step reaction mechanism in which a D^•^ is generated first and then reacts with H_2_O. When this DH_2_O^•^ reacts with DMPO according to Rxn 5, it can donate one hydrogen atom to DMPO from either two H atoms or one D atom. As a result, it would have a ∼2/3 probability of producing DMPO/H and a 1/3 probability of producing DMPO/D. Hence, this H_3_O^•^ hypothesis neatly explains the DMPO spin-trap experimental observations.

The existence of H_3_O^•^ was proposed more than 50 years ago^22^ and has since been a subject of ab initio modeling.^23−26^ However, the experimental evidence of this radical is still highly limited.^27−30^ Due to the highly complicated electrochemical environment in FCs, it would be nearly impossible to observe this radical directly in FCs, other than via the isotope substitution method as reported by Schlick et al.^6^ Here, we use ab initio modeling to investigate the properties of this radical when interacting with sulfonic anions and its role in PFSA membrane degradation.

We first performed a benchmarking study to search for optimal ab initio methods that balance the accuracy and computation speed because in the later stages of this study, we perform ab initio calculations with more than 200 electrons. Here, we use the H_3_O^•^...(H_2_O)_3_ cluster (model compound **2** in Figure 2) as the model system.^25,26^ Its smaller size enables us to perform CCSD (coupled-cluster singles-and-doubles)^32^ level of calculations with the aug-cc-pVTZ basis set, the result of which is used as the benchmark. We also performed calculations with MP2 and four different DFT methods. The formation energies Δ*E* under vacuum for **2** using different ab initio methods and basis sets then can be computed based on
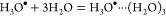
6

**Figure 2:**
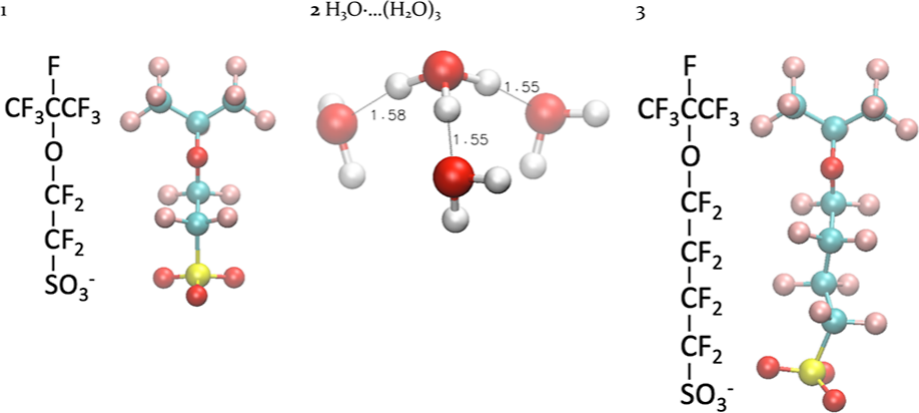
Model compounds used in this manuscript and their ground-state structures, optimized using ωB97XD/6-311++G(2d,p) under vacuum. The “···” in panel **2** means that this cluster is formed by hydrogen bonds. The structures presented in this manuscript were all generated using VMD^31^ with the color scheme: C: Cyan; H: White; O: Red; F: Ochre; and S: Yellow. The distances between atoms shown in this manuscript are all in angstroms.

The results are presented in Table 2. Compared with the CCSD benchmark, we conclude that MP2 and ωB97XD methods have higher accuracy, and the basis set only has a small impact on the calculation result. Therefore, we chose the ωB97XD method and the 6-311++G(2d,p) basis set to be used in the following stages due to the faster computational speed than the MP2 method.

**Table 2:** Reaction Energy (Δ*E*) Values in kcal/mol for Rxn 6 Calculated Using Different Ab Initio Methods and Basis Sets under Vacuum

	Basis Sets		
Methods	6-311++G(2d,p)	aug-cc-pVDZ	aug-cc-pVTZ
B3LYP	6.8	7.4	6.6
ωB97XD	14.3	15.0	13.2
M062X	9.5	9.9	8.9
B97D3	7.7	8.0	6.9
MP2	16.9	17.2	15.6
CCSD	16.0	16.0	14.1

In the next sections, we will discuss the H^•^ reacting with one water (H_2_O) molecule or a small water cluster associated with the sulfonic anion group (SO_3_^–^) of PFSA. The model compound **3** (Figure 2) used in the following steps represents one 3M ionomer side chain with a three-carbon backbone. The ground-state structure is shown in Figure 2 with a near-straight side chain. All calculations in the following sections are done under vacuum.

## 2 Results and Discussion

### 2.1 H^•^ Reactions without H_2_O

H^•^ can attack the *t*-F atom of **3** according to Rxn 2, or it can attack one of O atoms in SO_3_^–^ according to Rxn 4. For Rxn 2, Δ*E*^≠^ = 31.5 kcal/mol. The TS structure for this reaction is shown in Figure S1A, and the intrinsic reaction coordinate (IRC) calculation result is shown in Movie S1. For Rxn 4, Δ*E*^≠^ = 16.9 kcal/mol, and the TS structure is shown in Figure S1B. Thus, Rxn 4 is the preferred pathway when there is no H_2_O.

### 2.2 H^•^ Reacting with One H_2_O Molecule and Forming H_3_O^•^

Although H^•^ can react with H_2_O and form H_3_O^•^, H_3_O^•^ is not an energy-favorable compound because Rxn 7 has a Δ*E* value of 19.3 kcal/mol and a Δ*E*^≠^ value of 21.1 kcal/mol.
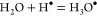
7

**Figure 3:**
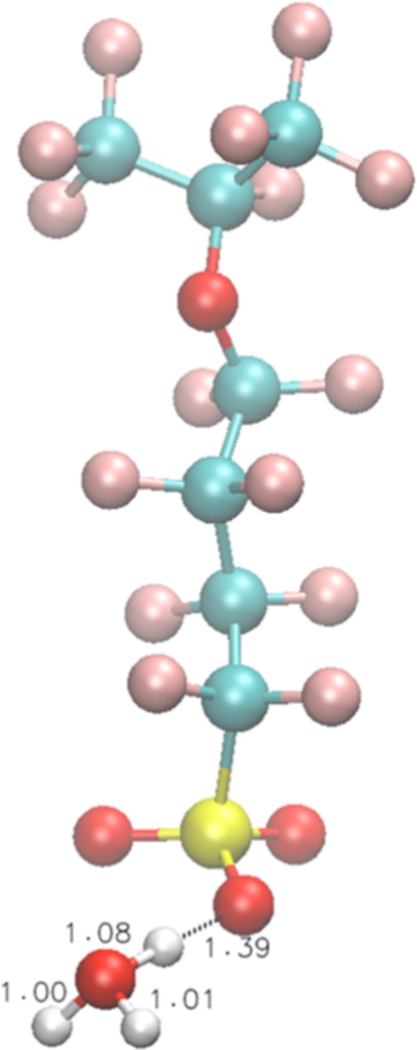
Optimized structures of **3** interacting with one H_3_O^•^ under vacuum.

However, the SO_3_^–^ group of **3** can form a stable intermediate state with H_3_O^•^ under vacuum via a reaction between H^•^ and an H_2_O molecule associating with SO_3_^–^ (Figure 3):
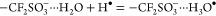
8

For Rxn 8, Δ*E* = 10.7 kcal/mol and Δ*E*^≠^ = 12.7 kcal/mol, indicating that SO_3_^–^ can provide 19.3–10.7 = 8.6 kcal/mol stabilization for H_3_O^•^.

We also found that the widely used PCM solvation model cannot produce any stable interaction structures for SO_3_^–^ and H_3_O^•^. During the geometry optimization, H_3_O^•^ will automatically decompose into H_2_O and H^•^ (Figure S2). Therefore, in this manuscript, we only perform vacuum calculations with one or more explicit water molecules, instead of using solvation models. Indeed, this vacuum calculation directly simulates dry conditions during FC operation, under which PEM degradation is found to be more significant. When one explicit water molecule is included, the ratio of water molecules to SO_3_^–^ groups (λ) is 1 and roughly corresponds to 10∼20% relative humidity (RH), and including three explicit water molecules corresponds to λ = 3 and 35∼40% RH.^3,33^

H^•^ can attack the *t*-F atom of **3** with the presence of one water molecule according to Rxn 9
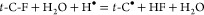
9

**Figure 4:**
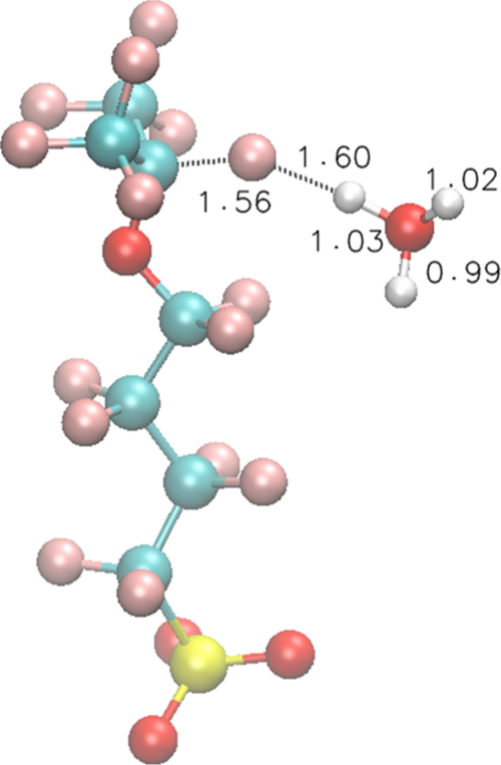
TS structures of Rxn 9 for model compound **3**.

Here, we assume that the side chain remains in a near-straight conformation, as in the structure of **3** in Figure 2. IRC calculation shows that H^•^ first reacts with H_2_O and then one of H atoms from H_2_O attacks the *t*-F, meaning that the H_2_O in Rxn 9 acts as a catalyst (Movie S2). In the TS, an H_3_O^•^ structure is formed (Figure 4). The overall Δ*E*^≠^ is 30.2 kcal/mol, which is 1.3 kcal/mol lower than that of Rxn 2, due to the fact that the H_3_O^•^ can provide some solvation for the TS.

**Figure 5:**
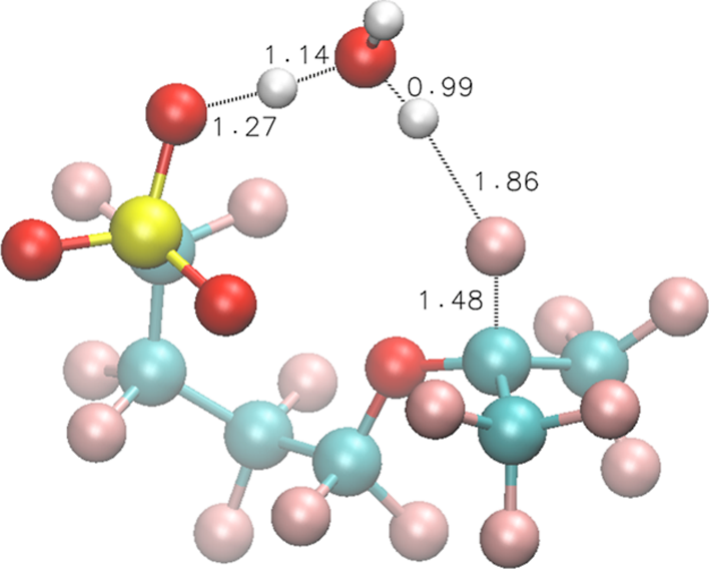
TS structures of Rxn 10 for model compound **3**.

As SO_3_^–^ can stabilize H_3_O^•^, one H_3_O^•^ associated with SO_3_^–^ may attack the *t*-F with lower Δ*E*^≠^. For such an attack, the side chain must undergo a conformational change so that SO_3_^–^ moves closer to the *t*-F (Figure 5 and IRC Movie S3)
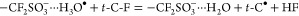
10

The Mulliken spin for the TS structure in Figure 5 is shown in Figure S3, indicating that the spin is mostly located on the H_3_O^•^. For Rxn 10, Δ*E*^≠^ = 10.4 kcal/mol, which is 0.5 kcal/mol smaller than that of Rxn 9. Because of the stabilization from SO_3_^–^, by combining Rxn 8 and Rxn 10, we have
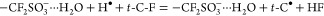
11

The overall Δ*E*^≠^ = 10.4 + 10.7 = 21.1 kcal/mol, significantly lower than that of Rxn 2 (31.5 kcal/mol) or Rxn 9 (30.2 kcal/mol) by 9∼10 kcal/mol, which is close to the stabilization energy (8.6 kcal/mol) provided by the −CF_2_SO_3_^–^...H_3_O^•^ interaction, suggesting that the lower TS barrier in Rxn 11 is mostly coming from the ion-radical stabilization effect. This H_3_O^•^ stabilized by SO_3_^–^ is also able to attack the secondary and primary F atoms (Figure S4); however, we found their Δ*E*^≠^ values to be 32.7 and 33.5 kcal/mol, respectively, much higher than that of *t*-F.

H_3_O^•^ can also attack an O atom of SO_3_^–^ (Figure S5)
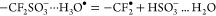
12Its Δ*E*^≠^ = 9.8 kcal/mol. By combining Rxn 8 with Rxn 13, we have
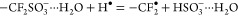
13

Then, the overall Δ*E*^≠^ = 10.7 + 9.8 = 20.5 kcal/mol.

The Δ*E*^≠^ values are summarized in Table 3. For the H^•^ reaction with one H_2_O, the attack at SO_3_^–^ still has the lowest overall Δ*E*^≠^ (Rxn 13). However, with the H_2_O acting as a catalyst and the stabilization provided by SO_3_^–^, Δ*E*^≠^ of the *t*-F attack is reduced significantly and gets closer to that of the H^•^ attacking at SO_3_^–^.

**Table 3:** Summary for Δ*E*^≠^ Values in kcal/mol for Reactions of Model Compound **3** Computed Using the ωB97XD Method and the 6-311++G(2d,p) Basis Set under Vacuum^a^

λ	Reactions		Δ*E*^≠^	TS Structures
0	(2)	*t*-C-F + H^•^ = *t*-C^•^ + HF	31.5	Figure S1A
	**(4)**	**–CF_2_SO_3_^–^ + H^•^ = −CF_2_^•^ + HSO_3_^–^**	**16.9**	**Figure S1B**
				
1	(9)	*t*-C-F + H_2_O + H^•^ = *t*-C^•^ + HF + H_2_O	30.2	Figure 4
	(11)	–CF_2_SO_3_^–^···H_2_O + H^•^ + *t*-C-F = −CF_2_SO_3_^–^···H_2_O + *t*-C^•^ + HF	21.1	Figure 5
	**(13)**	**–CF_2_SO_3_^–^···H_2_O + H^•^ = −CF_2_^•^ + HSO_3_^–^···H_2_O**	**20.5**	**Figure S5**
				
**2**	**(14)**	**–CF_2_SO_3_^–^···2H_2_O + H^•^ + *t*-C-F = −CF_2_SO_3_^–^...2H_2_O + *t*-C^•^ + HF**	**19.0**	**Figure 6**
	(15)	–CF_2_SO_3_^–^···2H_2_O + H^•^ = −CF_2_^•^ + HSO_3_^–^···2H_2_O	20.4	Figure S6B
				
**3**	**(16)**	**–CF_2_SO_3_^–^···3H_2_O + H^•^ + *t*-C-F = −CF_2_SO_3_^–^···3H_2_O + *t*-C^•^ + HF**	**21.0**	**Figure 7**
	(17)	–CF_2_SO_3_^–^···3H_2_O + H^•^ = −CF_2_^•^ + HSO_3_^–^···3H_2_O	24.9	Figure S7C

a The rows in bold are the preferred pathways for scenarios with different numbers of H_2_O molecules.

### 2.3 H^•^ Reacting with Two H_2_O Molecules

**Figure 6:**
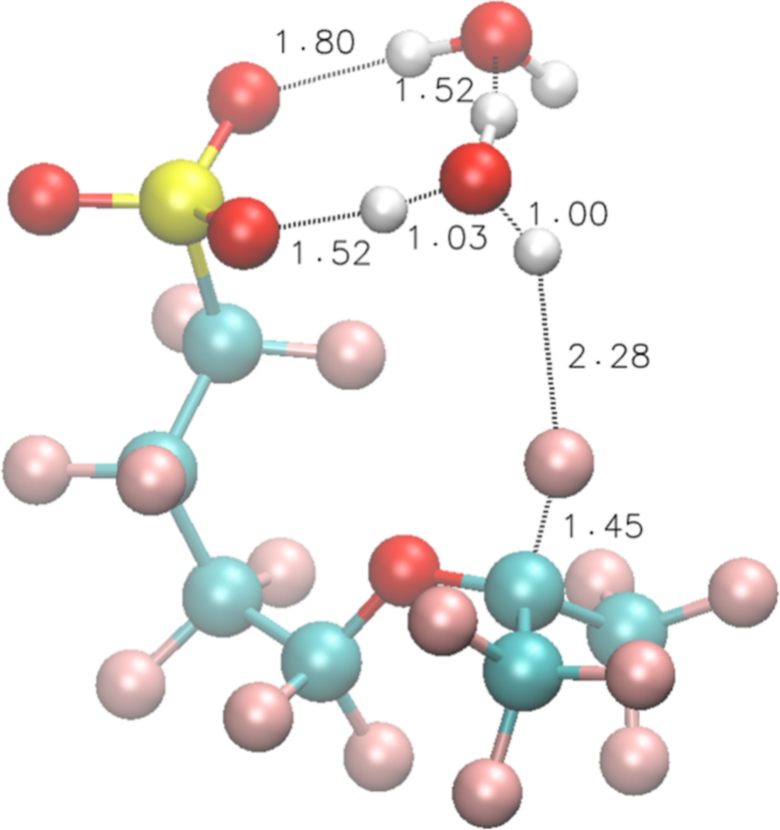
TS structures of Rxn 14 for model compound **3**.

When an H^•^ reacts with one of the two H_2_O molecules associated with SO_3_^–^ and forms an SO_3_^–^··· H_3_O^•^···H_2_O cluster (the optimized interacting structure is shown in Figure S6A), Δ*E* for this reaction is 8.1 kcal/mol, and Δ*E*^≠^ = 10.5 kcal/mol. If the side chain undergoes a conformational change, allowing the associated H_3_O^•^ to attack the *t*-F (Figure 6), Δ*E*^≠^ = 10.9 kcal/mol, leading to an overall Δ*E*^≠^ = 8.1 + 10.9 = 19.0 kcal/mol for Rxn 14.
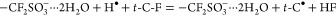
14

If H_3_O^•^ attacks an O atom of SO_3_^–^ (Rxn 15, the TS structure is shown in Figure S6B), Δ*E*^≠^ = 12.3 kcal/mol, resulting in an overall Δ*E*^≠^ = 8.1 + 12.3 = 20.4 kcal/mol.
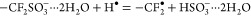
15

In this scenario, the preferred pathway with the lowest reaction barrier is the *t*-F degradation pathway.

### 2.4 H^•^ Reacting with Three H_2_O Molecules

**Figure 7:**
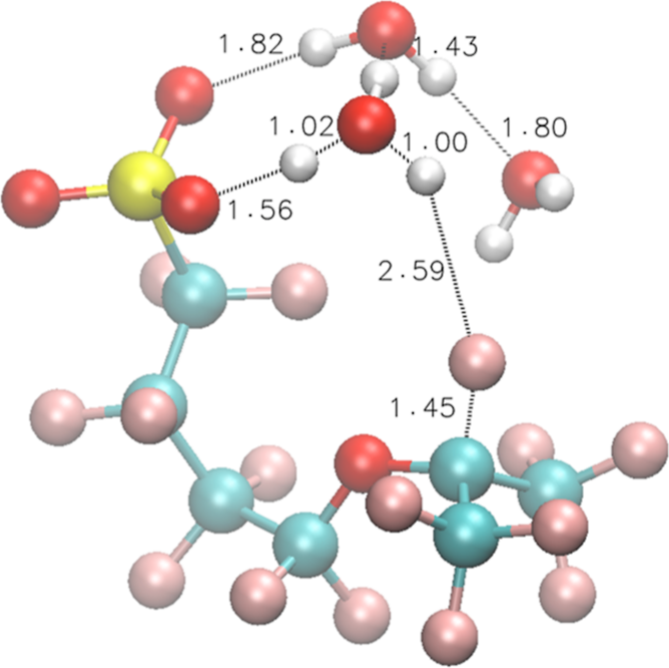
TS structures of Rxn 16 for model compound **3**.

When an H^•^ reacts with one of the three H_2_O molecules associated with the SO_3_^–^ and forms an SO_3_^–^···H_3_O^•^···2H_2_O cluster (the optimized interacting structure is shown in Figure S7A), Δ*E* for this reaction is 10.9 kcal/mol and Δ*E*^≠^ = 12.6 kcal/mol. This Δ*E* is slightly higher than the two-water scenario due to the cage-like structure formed by the three H_2_O molecules around SO_3_^–^ (Figure S7B), which stabilizes the reactant more and makes the Δ*E*^≠^ higher. If the side chain undergoes a conformational change, allowing the associated H_3_O^•^ to attack the *t*-F (Figure 7), Δ*E*^≠^ = 10.1 kcal/mol, leading to an overall Δ*E*^≠^ = 10.9 + 10.1 = 21.0 kcal/mol for Rxn 16.
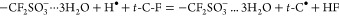
16

If H_3_O^•^ attacks an O atom of SO_3_^–^ (Rxn 17, the TS structure is shown in Figure S7C), Δ*E*^≠^ = 14.0 kcal/mol, resulting in an overall Δ*E*^≠^ = 10.9 + 14.0 = 24.9 kcal/mol.
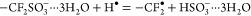
17

In this scenario, the preferred pathway with the lowest reaction barrier is still the *t*-F degradation pathway.

**Figure 8:**
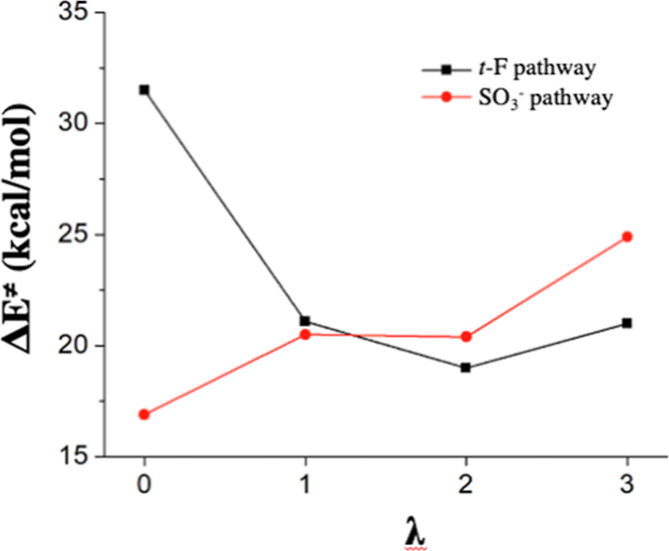
ΔE^≠^ values for *t*-F and SO_3_^–^ attacking reactions at different λ.

We have tried scenarios with more water molecules; however, because water molecules are not covalently bonded to PFSA, TS geometry optimization with so many loosely bonded water molecules failed to converge. Nevertheless, based on the above four scenarios, we can confidently conclude that the formation of H_3_O^•^ around SO_3_^–^ can lead to greatly reduced Δ*E*^≠^ for the *t*-F attack. In this mechanism, the water molecules around SO_3_^–^ not only provide solvation for the charged group but also act as a catalyst. In general, the *t*-F attack barrier decreases with more explicit water molecules, and the SO_3_^–^ attack barrier increases, making the former the preferred H^•^ degradation pathway at higher humidity (Figure 8). Our H_3_O^•^ hypothesis can explain not only previous isotopic substitution experiments^6^ but also why the *t*-F degradation reaction is a highly plausible H^•^ degradation mechanism for PEMs.^8,21^ To our knowledge, this is the first suggestion that H_3_O^•^ could be present in electrochemical devices with both experimental and theoretical support. The H_3_O^•^ may be directly generated from the hydrogen oxidation reaction (HOR), which could provide insights into the HOR mechanism as well. In addition, because perfluoroalkyl substances are widely detected in surface water and are notoriously difficult to break down, this work could also shed light on perfluoroalkyl removal in radical-based water treatment.^34^

## 3 Concluding Remarks

Two key discoveries of this work are (i) H_3_O^•^ stabilization by anions and (ii) the importance of the side chain conformational change. While we have proposed a plausible and thermodynamically sound reaction mechanism invoking the interaction of H^•^/H_3_O^•^ with PFSA ionomers, we should also note that Rxn 7 and 8 both indicate that H_3_O^•^ is not an energy-favorable compound. Therefore, it is not the lowest energy structure when H^•^ interacting with water clusters.^35^ One unanswered question in this manuscript is how H_3_O^•^ could be a stable radical and detected experimentally. We have performed some preliminary calculations showing that H_3_O^•^ could become energy favorable when interacting with multiple SO_3_^–^ anions and explicit water molecules, which will be the topic of our future report.

In addition, we also recognize that the proposed reaction pathway is likely to be kinetically challenged in the presence of O_2_ found in an operating fuel cell. H^•^ is known to react with O_2_ at a diffusion-controlled rate to form the benign HOO^•^.^36,37^ Thus, in the presence of O_2_, H^•^ or H_3_O^•^ would be quenched via this fast reaction. On the other hand, the HO^•^ radical can be formed by decomposing of H_2_O_2_ on the fuel cell electrodes and will attack to the PFSA by Rxn 3. Its Δ*E*^≠^ is around 22 kcal/mol for vacuum calculation at the ωB97XD/6-311++G(2d,p) level (please note that the values reported in Table 2 are computed with implicit solvation models). In addition, Yamaguchi et al. also reported that the HO^•^ attack barriers at the ether O will be reduced to ∼20 kcal/mol, also comparable to our barriers for H^•^ or H_3_O^•^ attack.^14^ Therefore, considering the similarity of H^•^ and HO^•^ reaction barriers and the availability of H^•^ or H_3_O^•^, at this stage we still cannot conclude which reaction would be the dominant degradation pathway. Nevertheless, it would be expected that ions such as SO_3_^–^, H^+^, and OH^–^ (in alkaline exchange membrane) might have similar stabilization or destabilization effect on H^•^, HO^•^, and HOO^•^ radicals, leading to enhanced or reduced membrane degradation barriers. This has mostly been neglected in previous computational modeling efforts for PFSA degradation. We are currently performing more DFT calculations to investigate whether interactions between HO^•^ and ions would lead to significantly reduced degradation reaction barriers.

The side chain conformational change can bring H_3_O^•^ closer to the *t*-F atom and facilitate the degradation reaction. This conformational change has also been neglected in previous modeling efforts. However, during the preparation of this manuscript, Yamaguchi et al. also reported that a similar HO^•^ interaction with SO_3_^–^ as well as a conformational change of side chain can greatly reduce the reaction barrier for the HO^•^ attack in PFSA.^14^ Thus, we believe that the interaction between SO_3_^–^ and radicals caused by the side chain conformational change should also be the focus in future PFSA degradation studies. In this manuscript, we did not model Nafion and Solvay ionomers. However, we believe that Nafion may undergo a similar conformational change, and the Solvay ionomer may not need significant conformational change for the interaction between SO_3_^–^ and *t*-F due to its shorter side chain (Figure 1). Based on this conformational change hypothesis, it is possible to enhance the degradation barrier by introducing steric interference in the TS structure via chemical substitutions, allowing for the design of PEMs with more durability. In addition, this work, as well as Yamaguchi et al.’s work,^14^ only considered intramonomer interactions between the reaction site and SO_3_^–^ of its own side chain. In PEMs, it is also possible to have intermonomer or even intermolecular interactions for an H_3_O^•^ associated with the SO_3_^–^ to attack any *t*-F nearby, which could be another future research direction.

Finally, from this work as well as our preliminary result for other radical/ion interactions, we have found that TS structures and energies obtained from vacuum calculations with explicit water molecules are dramatically different with those from implicit solvation model calculations. It is possible that either the current implicit solvation model may not perform well on radical reactions, or the implicit solvation model only corresponds to a fully solvated scenario in the bulk water.

## 4 Computational Methods

We used Gaussian 16C (G16C)^38^ to optimize the reactants, products, and TS structures. Every TS structure reported in this manuscript has been confirmed to have only one large imaginary frequency (Table S1). For a given reaction, Δ*E* or Δ*E*^≠^ values were obtained by comparing the total energy of the ground states of reactants with the total energy of the ground states of products or the energy of the TS state, respectively. No symmetry and dispersion correction were used during calculations.

We did not include the ZPVE in the computed energies in this manuscript for the following reasons. First, for higher level calculations such as CCSD and/or calculations with a larger basis set, the computational expense for extra ZPVE calculations is extremely high. Second, in some cases, the TS energy will be lower than the reactants’ energy when ZPVE is included. For example, Δ*E* and Δ*E*^≠^ values without ZPVE for Rxn 7 are 19.3 and 21.1 kcal/mol, respectively. However, when the ZPVE correction is added, they become 21.7 and 21.6 kcal/mol, respectively. Although introducing a scaling factor for ZPVE might solve this problem, it is beyond the scope of this manuscript.^39^ Finally, and the most importantly, we performed limited ZPVE calculations for some key reactions and found that including the ZPVE correction did not alter the relative energy barrier height for different reaction pathways, meaning that the inclusion of ZPVE would have no impact on our discussion or conclusions.

We also did not include the basis set superposition error (BSSE) correction in the computed energies. As shown in Table 2, the basis set only has a small impact on the calculation result, especially for DFT methods. BSSE calculations for Rxn 8 with the ωB97XD/6-311++G(2d,p) method resulted in a BSSE correction energy of only 0.05 kcal/mol.

## Supporting Information Available

Ground state and TS structures for select reactions (PDF)
IRC calculation S1 (MP4)
IRC calculation S2 (MP4)
IRC calculation S3 (MP4)

## Abbreviations

BSSE: basis set superposition error
CCSD: coupled-cluster singles and doubles
DMPO: 5,5-dimethylpyrroline *N*-oxide
DFT: density functional theory
FC: fuel cell
IRC: intrinsic reaction coordinate
HOR: hydrogen oxidation reaction
MP2: second order Møller–Plesset perturbation theory
PCM: polarizable continuum model
PEM: proton-exchange membrane
PFSA: perfluorosulfonic acid
RH: relative humidity
SMD: solvation model based on density
TS: transition state
*t*-F: tertiary fluorine
ZPVE: zero-point vibrational energy correction
Δ*E*^≠^: reaction barrier
λ: the ratio of water molecules to SO_3_^–^ groups

## References

[ref1] Schmittinger W.; Vahidi A. A Review of the Main Parameters Influencing Long-Term Performance and Durability of PEM Fuel Cells. J. Power Sources 2008, 180, 1–14. 10.1016/j.jpowsour.2008.01.070.

[ref2] Zatoń M.; Rozière J.; Jones D. J. Current Understanding of Chemical Degradation Mechanisms of Perfluorosulfonic Acid Membranes and Their Mitigation Strategies: A Review. Sustain. Energy Fuels 2017, 1, 409–438. 10.1039/C7SE00038C.

[ref3] Kusoglu A.; Weber A. Z. New Insights into Perfluorinated Sulfonic-Acid Ionomers. Chem. Rev. 2017, 117, 987–1104. 10.1021/acs.chemrev.6b00159. 28112903

[ref4] Wang Y.; Ruiz Diaz D. F.; Chen K. S.; Wang Z.; Adroher X. C. Materials, technological status, and fundamentals of PEM fuel cells - A review. Mater. Today 2020, 32, 178–203. 10.1016/j.mattod.2019.06.005.

[ref5] Cullen D. A.; Neyerlin K. C.; Ahluwalia R. K.; Mukundan R.; More K. L.; Borup R. L.; Weber A. Z.; Myers D. J.; Kusoglu A. New Roads and Challenges for Fuel Cells in Heavy-Duty Transportation. Nat. Energy 2021, 6, 462–474. 10.1038/s41560-021-00775-z.

[ref6] Danilczuk M.; Coms F. D.; Schlick S. Visualizing Chemical Reactions and Crossover Processes in a Fuel Cell Inserted in the ESR Resonator: Detection by Spin Trapping of Oxygen Radicals, Nafion-Derived Fragments, and Hydrogen and Deuterium Atoms. J. Phys. Chem. B 2009, 113, 8031–8042. 10.1021/jp901597f. 19453175

[ref7] Lin L.; Danilczuk M.; Schlick S. Electron Spin Resonance Study of Chemical Reactions and Crossover Processes in a Fuel Cell: Effect of Membrane Thickness. J. Power Sources 2013, 233, 98–103. 10.1016/j.jpowsour.2013.01.117.

[ref8] Yandrasits M. A.; Marimannikkuppam S.; Lindell M. J.; Kalstabakken K. A.; Kurkowski M.; Ha P. Ion Chromatography and Combustion Ion Chromatography Analysis of Fuel Cell Effluent Water During Open Circuit Voltage. J. Electrochem. Soc. 2022, 169, 034526 10.1149/1945-7111/ac5d96.

[ref9] Coms F. D. The Chemistry of Fuel Cell Membrane Chemical Degradation. ECS Trans. 2008, 16, 235 10.1149/1.2981859.

[ref10] Yu T. H.; Sha Y.; Liu W.-G.; Merinov B. V.; Shirvanian P.; Goddard W. A. Mechanism for Degradation of Nafion in PEM Fuel Cells from Quantum Mechanics Calculations. J. Am. Chem. Soc. 2011, 133, 19857–19863. 10.1021/ja2074642. 22017316

[ref11] Kumar M.; Paddison S. J. Side-Chain Degradation of Perfluorosulfonic Acid Membranes: An Ab Initio Study. J. Mater. Res. 2012, 27, 1982–1991. 10.1557/jmr.2012.191.

[ref12] Zhao Y.; Yamaguchi M.; Tsuchida E.; Choe Y.-K.; Ikeshoji T. DFT Studies of Perfluorosulfonic Acid Ionomer Degradation in Fuel Cells. J. Phys. Chem. C 2018, 122, 20135–20143. 10.1021/acs.jpcc.8b05908.

[ref13] Yamaguchi M. DFT Study on the Chemical Degradation Mechanism of Perfluorobis(Sulfonyl)Imide Sulfonic Acid Ionomer Membranes. J. Phys. Chem. C 2021, 125, 1929–1939. 10.1021/acs.jpcc.0c08105.

[ref14] Yamaguchi M. DFT Study on Side Chain Detachment of Perfluorosulfonic Acid Ionomers by Radical-Assisted Nucleophilic Attack of Water. Polym. Degrad. Stab. 2022, 196, 109832 10.1016/j.polymdegradstab.2022.109832.

[ref15] Bajaj A.; Liu F.; Kulik H. J. Uncovering Alternate Pathways to Nafion Membrane Degradation in Fuel Cells with First-Principles Modeling. J. Phys. Chem. C 2020, 124, 15094–15106. 10.1021/acs.jpcc.0c04417.

[ref16] Møller C.; Plesset M. S. Note on an Approximation Treatment for Many-Electron Systems. Phys. Rev. 1934, 46, 618–622. 10.1103/PhysRev.46.618.

[ref17] Tomasi J.; Mennucci B.; Cammi R. Quantum Mechanical Continuum Solvation Models. Chem. Rev. 2005, 105, 2999–3094. 10.1021/cr9904009. 16092826

[ref18] Marenich A. V.; Cramer C. J.; Truhlar D. G. Universal Solvation Model Based on Solute Electron Density and on a Continuum Model of the Solvent Defined by the Bulk Dielectric Constant and Atomic Surface Tensions. J. Phys. Chem. B 2009, 113, 6378–6396. 10.1021/jp810292n. 19366259

[ref19] Dreizler A. M.; Roduner E. Reaction Kinetics of Hydroxyl Radicals with Model Compounds of Fuel Cell Polymer Membranes. Fuel Cells 2012, 12, 132–140. 10.1002/fuce.201100157.

[ref20] Coms F. D.; Xu H.; McCallum T.; Mittelsteadt C. Mechanism of Perfluorsulfonic Acid Membrane Chemical Degradation Under Low RH Conditions. ECS Trans. 2013, 50, 907 10.1149/05002.0907ecst.

[ref21] Yandrasits M. A.; Komlev A.; Kalstabakken K.; Kurkowski M. J.; Lindell M. J. Liquid Chromatography/Mass Spectrometry Analysis of Effluent Water from PFSA Membrane Fuel Cells Operated at OCV. J. Electrochem. Soc. 2021, 168, 024517 10.1149/1945-7111/abe56a.

[ref22] Bernstein H. J. H. H Atom Adducts-New Free Radicals?. J. Am. Chem. Soc. 1963, 85, 484–485. 10.1021/ja00887a033.

[ref23] Niblaeus K. S. E.; Roos B. O.; Siegbahn P. E. M. Theoretical Studies on the Stability of the H3O Radical Based on Ab Initio UHF-CI Calculations. Chem. Phys. 1977, 25, 207–213. 10.1016/0301-0104(77)87077-8.

[ref24] McLoughlin P. W.; Gellene G. I. Ab Initio Investigation of Possible Dynamical Stabilization of the Oxonium Radical. J. Phys. Chem. 1992, 96, 4396–4404. 10.1021/j100190a051.

[ref25] Sobolewski A. L.; Domcke W. Hydrated Hydronium: A Cluster Model of the Solvated Electron?. Phys. Chem. Chem. Phys. 2002, 4, 4–10. 10.1039/b107373g.

[ref26] Uhlig F.; Marsalek O.; Jungwirth P. From a localized H3O radical to a delocalized H3O+···e– solvent-separated pair by sequential hydration. Phys. Chem. Chem. Phys. 2011, 13, 14003–14009. 10.1039/C1CP20764D. 21750807

[ref27] Martin T. W.; Swift L. L. Discovery and Electron Spin Resonance Spectra of Matrix-Stabilized Hydronium Radicals H3O and D3O. J. Am. Chem. Soc. 1971, 93, 2788–2790. 10.1021/ja00740a038.

[ref28] Poterya V.; Fárník M.; Slavíček P.; Buck U.; Kresin V. V. Photodissociation of Hydrogen Halide Molecules on Free Ice Nanoparticles. J. Chem. Phys. 2007, 126, 071101 10.1063/1.2709635. 17328585

[ref29] Poterya V.; Fedor J.; Pysanenko A.; Tkáč O.; Lengyel J.; Ončák M.; Slavíček P.; Fárník M. Photochemistry of HI on argon and waternanoparticles: Hydronium radical generation in HI·(H2O)n. Phys. Chem. Chem. Phys. 2011, 13, 2250–2258. 10.1039/C0CP01518K. 21116552

[ref30] Hernández F. J.; Capello M. C.; Naito A.; Manita S.; Tsukada K.; Miyazaki M.; Fujii M.; Broquier M.; Gregoire G.; Dedonder-Lardeux C.; Jouvet C.; Pino G. A. Trapped Hydronium Radical Produced by Ultraviolet Excitation of Substituted Aromatic Molecule. J. Phys. Chem. A 2015, 119, 12730–12735. 10.1021/acs.jpca.5b10142. 26637013

[ref31] Humphrey W.; Dalke A.; Schulten K. VMD: Visual molecular dynamics. J. Mol. Graph. 1996, 14, 33–38. 10.1016/0263-7855(96)00018-5. 8744570

[ref32] Scuseria G. E.; Janssen C. L.; Schaefer H. F. An efficient reformulation of the closed-shell coupled cluster single and double excitation (CCSD) equations. J. Chem. Phys. 1988, 89, 7382–7387. 10.1063/1.455269.

[ref33] Mittelsteadt C. K.; Liu H. Conductivity, Permeability, and Ohmic Shorting of Ionomeric Membranes. Handbook of Fuel Cells: Advances in Electrocatalysis, Materials, Diagnostics and Durability; Wiley & Sons, 2010; Vol. 5–6, pp 345–358.

[ref34] Singh R. K.; Fernando S.; Baygi S. F.; Multari N.; Thagard S. M.; Holsen T. M. Breakdown Products from Perfluorinated Alkyl Substances (PFAS) Degradation in a Plasma-Based Water Treatment Process. Environ. Sci. Technol. 2019, 53, 2731–2738. 10.1021/acs.est.8b07031. 30768259

[ref35] Alexandrova A. N. H·(H2O)n Clusters: Microsolvation of the Hydrogen Atom via Molecular ab Initio Gradient Embedded Genetic Algorithm (GEGA). J. Phys. Chem. A 2010, 114, 12591–12599. 10.1021/jp1092543. 21077611

[ref36] Buxton G. V.; Greenstock C. L.; Helman W. P.; Ross A. B. Critical Review of rate constants for reactions of hydrated electrons, hydrogen atoms and hydroxyl radicals (·OH/·O– in Aqueous Solution. J. Phys. Chem. Ref. Data 1988, 17, 513–886. 10.1063/1.555805.

[ref37] Coms F. D.; Schlick S.; Danilczuk M. Stabilization of Perfluorinated Membranes Using Ce3+and Mn2+Redox Scavengers. In The Chemistry of Membranes Used in Fuel Cells; John Wiley & Sons, Ltd, 2018, pp 75–106.

[ref38] Frisch M. J.; Trucks G. W.; Schlegel H. B.; Scuseria G. E.; Robb M. A.; Cheeseman J. R.; Scalmani G.; Barone V.; Petersson G. A.; Nakatsuji H.; Li X.; Caricato M.; Marenich A. V.; Bloino J.; Janesko B. G.; Gomperts R.; Mennucci B.; Hratchian H. P.; Ortiz J. V.; Izmaylov A. F.; Sonnenberg J. L.; Williams-Young D.; Ding F.; Lipparini F.; Egidi F.; Goings J.; Peng B.; Petrone A.; Henderson T.; Ranasinghe D.; Zakrzewski V. G.; Gao J.; Rega N.; Zheng G.; Liang W.; Hada M.; Ehara M.; Toyota K.; Fukuda R.; Hasegawa J.; Ishida M.; Nakajima T.; Honda Y.; Kitao O.; Nakai H.; Vreven T.; Throssell K.; Montgomery J. A. Jr.; Peralta J. E.; Ogliaro F.; Bearpark M. J.; Heyd J. J.; Brothers E. N.; Kudin K. N.; Staroverov V. N.; Keith T. A.; Kobayashi R.; Normand J.; Raghavachari K.; Rendell A. P.; Burant J. C.; Iyengar S. S.; Tomasi J.; Cossi M.; Millam J. M.; Klene M.; Adamo C.; Cammi R.; Ochterski J. W.; Martin R. L.; Morokuma K.; Farkas O.; Foresman J. B.; Fox D. J. Gaussian 16 Revision C.01; Gaussian Inc., 2016.

[ref39] Alecu I. M.; Zheng J.; Zhao Y.; Truhlar D. G. Computational Thermochemistry: Scale Factor Databases and Scale Factors for Vibrational Frequencies Obtained from Electronic Model Chemistries. J. Chem. Theory Comput. 2010, 6, 2872–2887. 10.1021/ct100326h. 26616087

