# Supplementary material for: Role of H3O· Radical in the Degradation of Fuel Cell Proton-Exchange Membranes
Source: ACS Phys Chem Au. 2022 Oct 18;2(6):527–34. doi: 10.1021/acsphyschemau.2c00037 (PMC9955370; doi:10.1021/acsphyschemau.2c00037)
Supplement: Supplementary file 1 — pg2c00037_si_001.pdf [file pg2c00037_si_001.pdf]

Supporting Information for

The Role of $H_3O\cdot$ Radical in the Degradation of Fuel Cell Proton Exchange Membranes

Hai Long[a,], Clara Larson[a], Frank Coms[b], Bryan Pivovar[c], Gregg Dahlke[d], and Michael Yandrasits[e]*

a. Computational Science Center, National Renewable Energy Laboratory, 15013 Denver West Parkway, Golden, CO 80401, United States

b. Global Fuel Cell Business, General Motors Company, 850 N Glenwood Avenue, Pontiac, MI 48340, United States

c. Chemical and Materials Science Center, National Renewable Energy Laboratory, 15013 Denver West Parkway, Golden, CO 80401, United States

d. 3M Advanced Materials Division Laboratory, 3M Center, Saint Paul, Minnesota 55144-1000, United States

e. 3M Corporate Research Materials Laboratory, 3M Center, Saint Paul, Minnesota 55144-1000, United States

Fig S1. TS structures of **3** (A) for Rxn 2 and (B) Rxn 4.

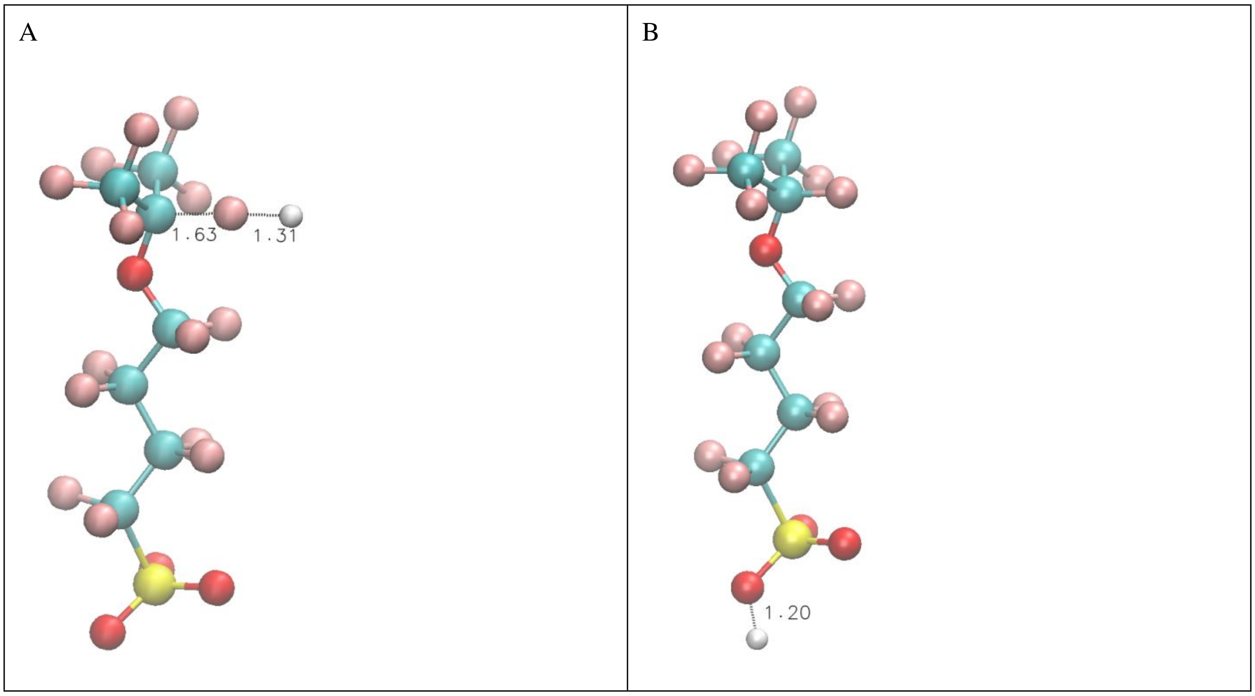

Fig S2: Structures of **3** interacting with one $H_3O\cdot$ in vacuum with PCM solvation model. The optimization fails to converge with one H· flying away and this figure shows the last snapshot of optimization.

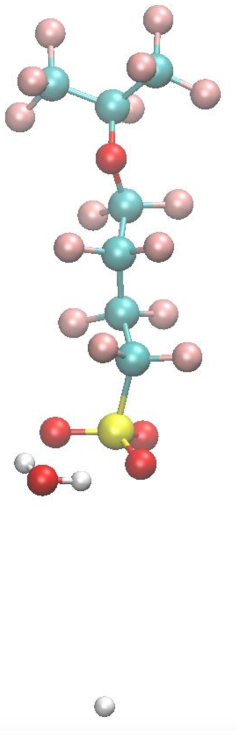

Fig S3: Mulliken spin for the TS structure in Fig 5.

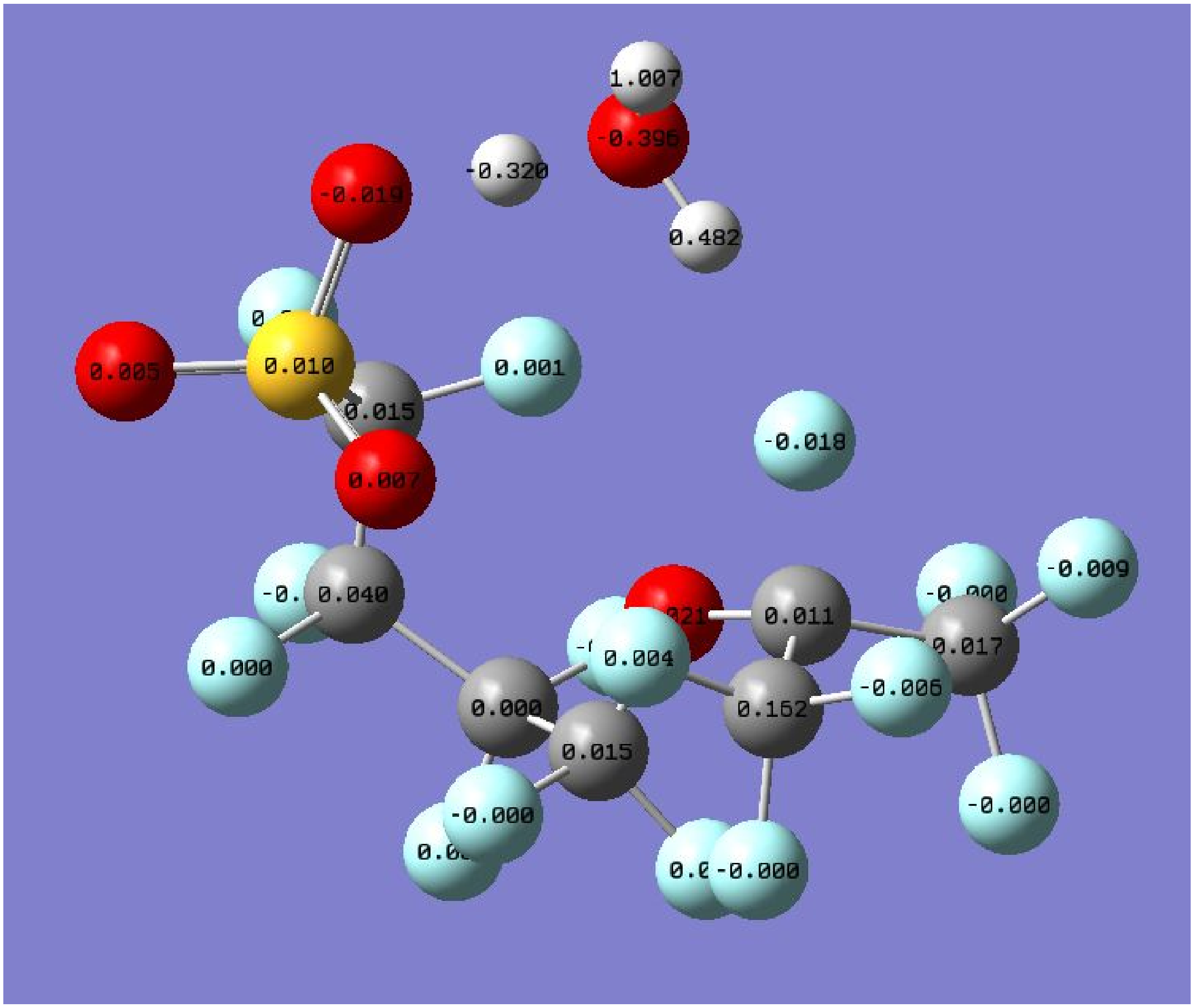

Fig S4. TS structures of a H_3O· stabilized by SO_3^- attacking to (A) the secondary and (B) primary F atoms. The overall ΔE≠ values are 32.7 and 33.5 kcal/mol, respectively.

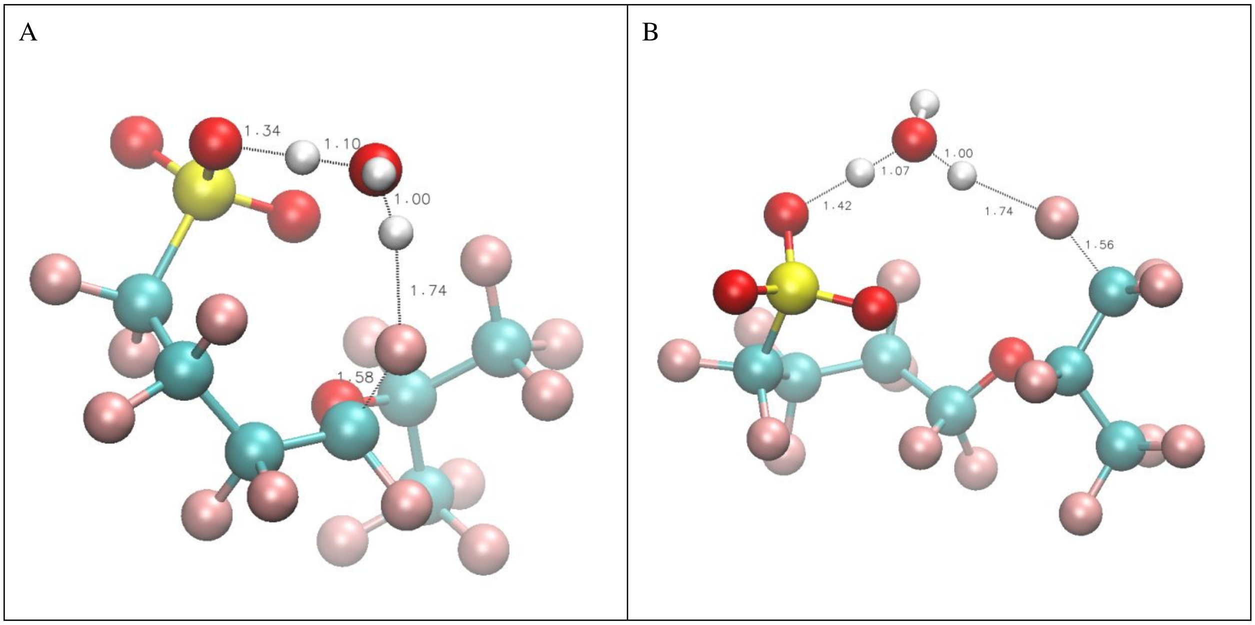

Fig S5. TS structures of Rxn 12 for model compound **3**.

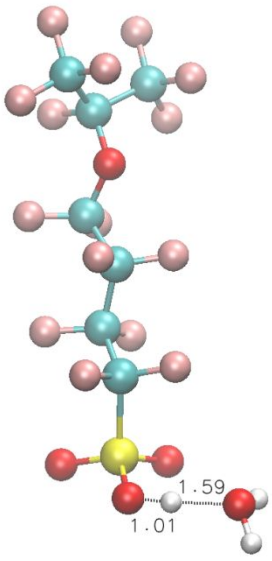

Fig S6. (A) Optimized interacting structure for the SO_3^-… H_3O·…H_2O cluster and (B) TS structure of Rxn 15.

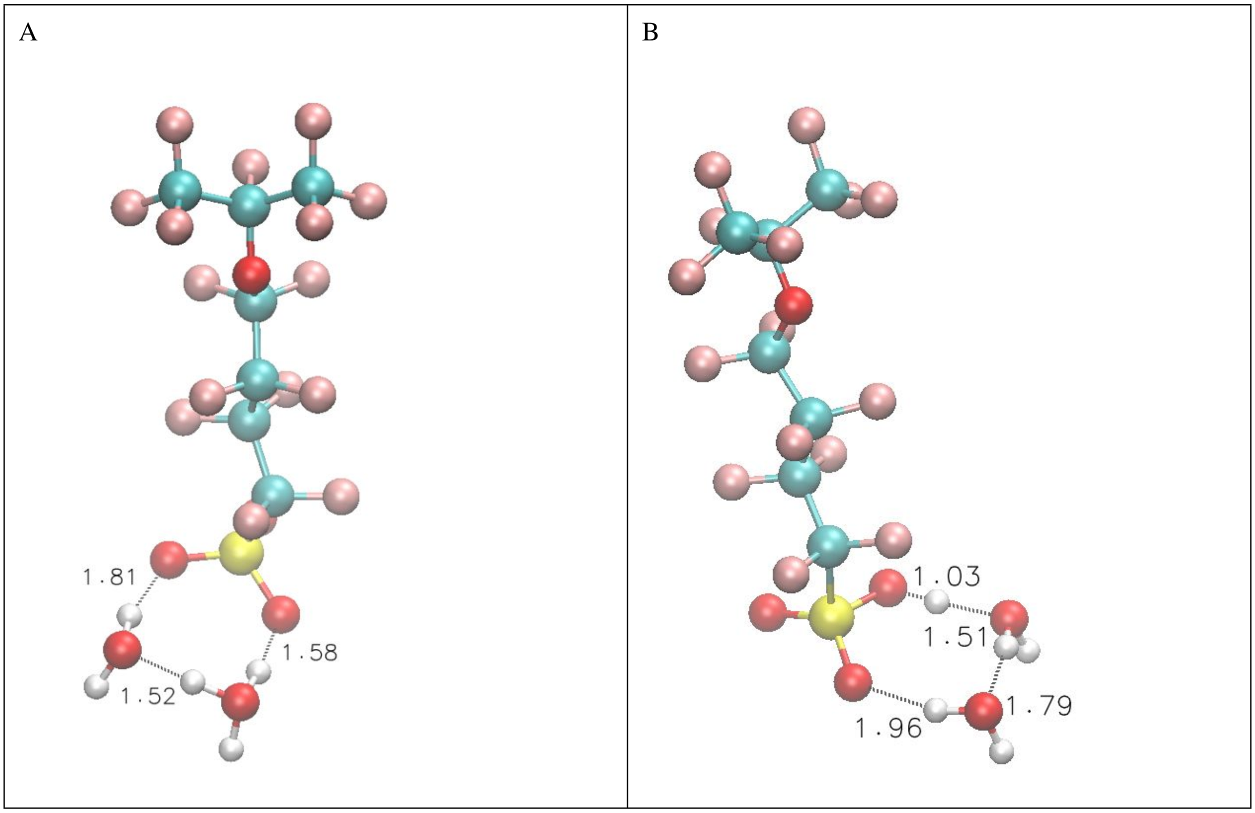

Fig S7. (A) Optimized interacting structure for the SO_3^-… H_3O·…$2H_2O$ cluster, (B) Optimized interacting structure for the SO_3^-…$3H_2O$ cluster and (C) TS structure of Rxn 17.

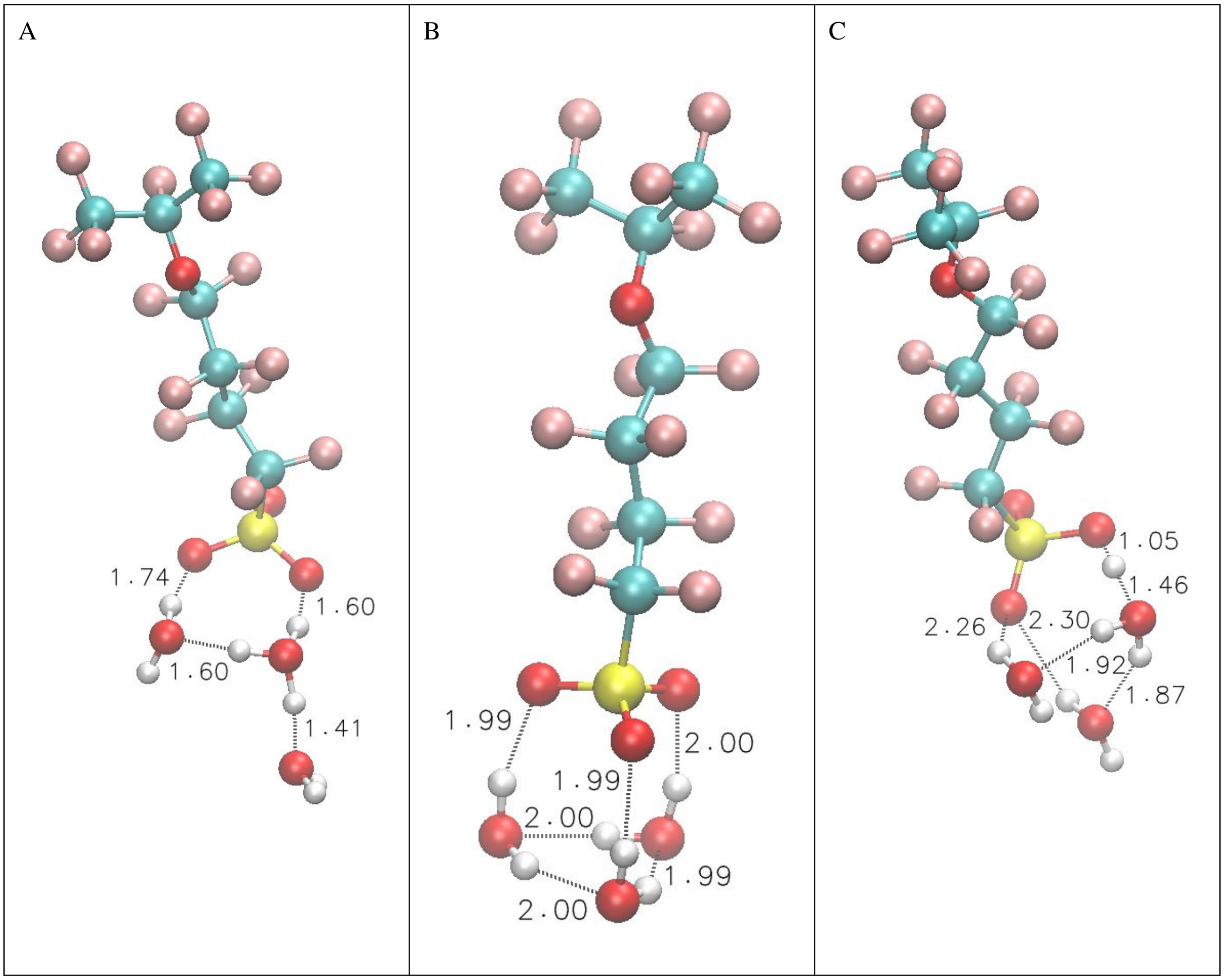

Table S1. Lowest nine vibrational frequencies from select TS calculations in cm^{-1}. As a convention of Gaussian G16, the imaginary frequency is reported as the "negative" frequency. For each TS structure, only one large negative frequency (< -10 cm^{-1}) is detected.

Structure	1	2	3	4	5	6	7	8	9
Fig S1	-1810.0495	-6.1833	-0.0012	-0.0010	0.0006	2.0837	3.3501	8.1911	22.7070
Fig 4	-1829.6972	-6.0617	-2.8425	-0.0008	0.0010	0.0015	2.0302	18.4970	22.4156
Fig 5	-1861.9963	-6.5771	-3.1420	0.0009	0.0012	0.0014	2.0288	23.1505	35.3716
Fig 6	-1413.3901	-5.7663	-2.8371	-0.9020	-0.0001	0.0004	0.0012	16.6878	23.4729
Fig 7	-1222.3071	-5.0921	0.0017	0.0019	0.0019	2.1847	3.1917	18.0101	29.3034
